# Structural and Optical Properties of Solvated PbI_2_ in γ-Butyrolactone: Insight into the Solution Chemistry of Lead Halide Perovskite Precursors

**DOI:** 10.1021/acs.jpclett.0c01890

**Published:** 2020-07-09

**Authors:** Eros Radicchi, Ali Kachmar, Edoardo Mosconi, Beatrice Bizzarri, Francesca Nunzi, Filippo De Angelis

**Affiliations:** † Department of Chemistry, Biology and Biotechnology, University of Perugia, via Elce di Sotto 8, 06123 Perugia, Italy; ‡ Computational Laboratory for Hybrid/Organic Photovoltaics (CLHYO), Istituto CNR di Scienze e Tecnologie Chimiche “Giulio Natta” (CNR-SCITEC), via Elce di Sotto 8, 06123 Perugia, Italy; § Qatar Environment and Energy Research Institute, Hamad Bin Khalifa University, P.O. Box 5825, Doha, Qatar; ∥ CompuNet, Istituto Italiano di Tecnologia, Via Morego 30, 16163 Genova, Italy

## Abstract

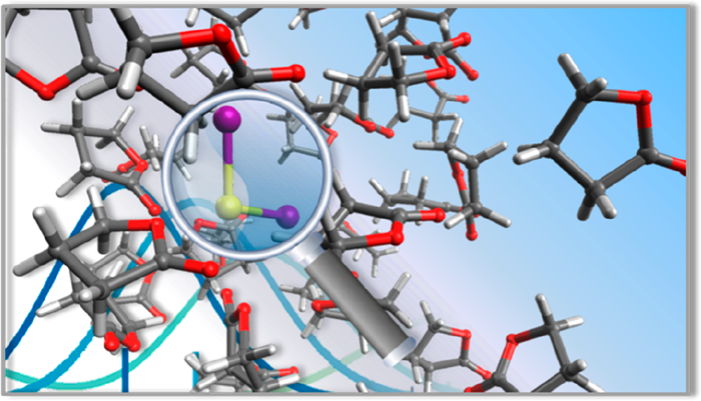

We employ a fine-tuned theoretical framework, combining *ab initio* molecular dynamics (AIMD), density functional theory (DFT), and time-dependent (TD) DFT methods, to investigate the interactions and optical properties of the iodoplumbates within the low coordinative γ-butyrolactone (GBL) solvent environment, widely employed in the perovskite synthesis. We uncover the extent of GBL coordination to PbI_2_ investigating its relation to the solvated PbI_2_ optical properties. The employed approach has been further validated by comparison with the experimental UV–vis absorption spectrum of PbI_2_ in GBL solvent. A comparison with other solvents, commonly employed in the perovskite synthesis, such as *N*,*N*-dimethylformamide (DMF) and dimethyl sulfoxide (DMSO) is also reported. The methodology developed in this work can be reasonably extended to the investigation of similar systems.

Lead-halide perovskites are one of the most studied materials in the last years, due to their very peculiar optoelectronic properties that have allowed their rapid application in widespread devices, such as solar cells and light emitting diodes.^1,2^ Several approaches have been developed to prepare perovskites with different properties. Among these, the solution synthesis is by far the easiest and most adopted method to synthesize these materials in research laboratories, involving different techniques, such as spin-coating or blade-coating for the deposition of thin films.^3−5^ Solvent engineering plays an important role in the nucleation and growth of perovskites, and in achieving high power conversion efficiency.^6−8^ Many researchers already proved that the choice of solvent is crucial for the morphology of solution-deposited perovskite films and thus for the final quality of devices. In particular, the interactions between individual PbX_2_(X = Cl, Br, I) precursors and halide salts in solution drive the formation of the perovskite during the deposition and the annealing processes, affecting the crystallization of the material and, consequently, the device performances.^5,9−14^ Moreover, solvent coordination influences the nature of species present in the solution environment, thus regulating the type and quantity of defects that are likely to be found in the crystalline material.^15−20^ Highly polar aprotic solvents are generally employed, such as dimethyl sulfoxide (DMSO), *N*,*N*-dimethylformamide (DMF), γ-butyrolactone (GBL), acetonitrile (ACN), or a combination thereof. The solubility of PbX_2_ and halide salts in these solvents or their mixtures was found to vary. In particular, GBL is a low solubility solvent for both precursors and MAPbX_3_ (MA = methylammonium) that is able to form single crystals of perovskite.^21,22^ Fateev et al.^23^ reported the formation of solid-state adducts upon evaporation of a concentrated perovskite solution in GBL environment, similarly to what happens in DMSO^6,7,24^ or DMF.^25,26^ Kadro et al. illustrated a facile method for rapid growth of large, freestanding crystals of MAPbI_3_ from a GBL solution through heating at unconventionally high temperatures.^27^ Rahimnejad et al.^15^ highlighted the formation of PbI_3_^–^ complex in diluted PbI_2_ solution and addressed it to the low nucleophilicity of GBL. Recently, Ahlawat et al.^28^ carried out simulations on the nucleation of MAPbI_3_ from a GBL solution, aiming to disclose the atomistic details of the perovskite crystallization process. Classical molecular dynamics simulations were also performed by Gutierrez-Sevillano et al.^29^ on perovskite precursors to study perovskite formation and stability. Despite the important progress in exploring the solution chemistry of lead halide perovskite precursors, a detailed knowledge of the interactions at the atomic level and how these reflect on the optical properties of the solvated PbI_2_ species are still lacking. Consequently, here we propose a computational protocol to study the behavior of the PbI_2_ precursor salt in the GBL solvent under non colloidal conditions, a behavior that is expected when dealing with diluted solutions. The nature of other species in the GBL solvent environment will be considered in a following paper. To uncover the interactions of the PbI_2_ system at the atomic level, we carried out *ab initio* molecular dynamics (AIMD) simulation of the solvated PbI_2_ complexes and we evaluated the energetics and the geometries of static PbI_2_(GBL)_*n*_ complexes by means of density functional theory (DFT) methods. We used the outcome of these calculations to simulate by means of time-dependent (TD) DFT methods the complexes absorption spectra, which we then compared with UV–vis spectroscopy experimental data, employing a combination of tools that was already successfully adopted for the understanding of the lead halide perovskite precursors optical properties.^18^ The fine-tuned methodology developed in this work can be reasonably extended to the investigation of similar systems.

To investigate the properties of the PbI_2_ GBL solution, in particular to identify the preferred coordination and to check the presence of specific interactions in the solution environment, we first carried out AIMD simulation of a PbI_2_ molecule in explicit bulk GBL solvent. To do this, we setup the simulation of the GBL solvent, we calculated the volume occupied by a single GBL molecule, and we ran a first short (∼5 ps) molecular dynamics simulation of an initial model with 4 GBL molecules. Taking the results of this simulation, we created a supercell containing 32 GBL molecules in a cubic cell with *a* = *b* = *c* = 15.94 Å and we ran a longer (10 ps) AIMD simulation, equilibrating the system at 300 K. On this equilibrated system we replaced one GBL molecule with a PbI_2_ molecule (see Figure 1a) and we ran again 20 ps of AIMD simulation. We then took average statistics, discarding the initial 5 ps of the thermalization.

**Figure 1:**
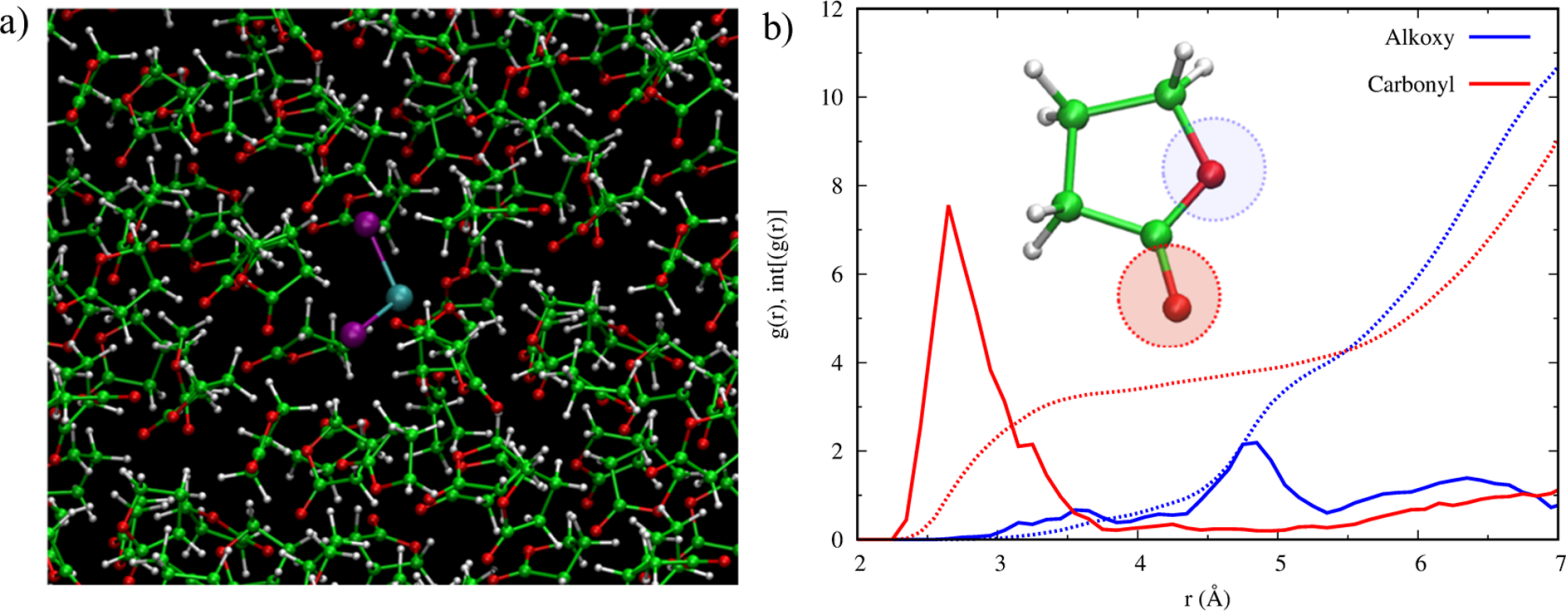
(a) Model of PbI_2_ in GBL solvent. (b) Radial pair distribution function, *g*(*r*) (continuous lines), and its integration, int[*g*(*r*)] (dotted lines), of the Pb–O distance (r, Å; red and blue lines correspond to carbonyl and alkoxy oxygens).

The outcome of this simulation gives us some interesting information on the structural properties of the solvated PbI_2_. First of all, we found that the PbI_2_ molecule did not dissociate during the dynamics, consistently with the low coordinative power of the GBL solvent. Opposite to this, in DMSO we experimentally found PbI_2_ to dissociate into PbI^+^/Pb^2+^ and I^–^, in line with the stronger coordinative power of this solvent.^18,30^ Moreover, the I–Pb–I angle always lies around 90° during the whole AIMD simulation (see Figure S1, Supporting Information), indicative that the initial equatorial PbI_2_ arrangement is retained throughout the dynamics.

To investigate the solvent coordination sphere around PbI_2_, we analyzed the Pb–O radial pair distribution function *g*(*r*) and its integral, int[*g*(*r*)], considering both the carbonyl and alkoxy oxygen atoms of GBL, Figure 1b. GBL mainly interacts with Pb through the carbonyl oxygen, with a distance of ∼2.7 Å and an average coordination number between 2 and 3, as shown by the value of int[*g*(*r*)] below 3 Å. We can also notice a broad and less structured feature related to the alkoxy O–Pb distance, which range starts at ∼3 Å and reaches higher values around ∼4.9 Å. The longer average bond distances and the less structured features are indicative of a weaker interaction between Pb and alkoxy oxygen, still a small fraction of GBL molecules features quite short alkoxy O–Pb bond lengths. The average Pb–O coordination number observed during the whole dynamics, evaluated considering only the GBL molecules directly bonded with Pb, confirms that PbI_2_ is bonded on average with two or three GBL molecules; see Figure S2, Supporting Information.

The overall *g*(*r*) tells us the average GBL coordination to Pb; however, it does not allow us to discriminate whether a single GBL molecule would individually or concomitantly bind Pb through the carbonyl and alkoxy oxygen atoms. To look closer at these interactions, we move to consider the individual *g*(*r*) for each of the 31 GBL molecules involved in our model; see Figure 2 and Figure S3, Supporting Information. While most of the molecules are noninteracting, with positive values of *g*(*r*) only above 4 Å or more, we identify five GBL molecules that are clearly bonded with the PbI_2_ molecule (see panels a–e in Figure 2). Interestingly, the considered solvent molecules do not interact with Pb in the same way: in two cases (panels a and b), the carbonyl oxygen is the only GBL atom effectively involved in the interaction with Pb, with *g*(*r*) peaks at 2.65 Å, representative of the average Pb–O coordination. Two other GBL molecules (panels c and d) still interact mainly through the carbonyl oxygen, but the alkoxy oxygen also shows a contribution, resulting in a pincher-type interaction. In this case, the carbonyl O–Pb interaction is weakened, with *g*(*r*) peaks at 2.95 and 3.05 Å against values of 2.65 Å in a and b cases, and *g*(*r*) peaks at 3.65 and 3.75 Å for the alkoxy O–Pb distances. Last, one GBL molecule (panel e) surprisingly shows interaction with PbI_2_ only by the alkoxy oxygen, with a *g*(*r*) peak at 3.45 Å, while the carbonyl oxygen lies distant from Pb, not effectively interacting.

**Figure 2:**
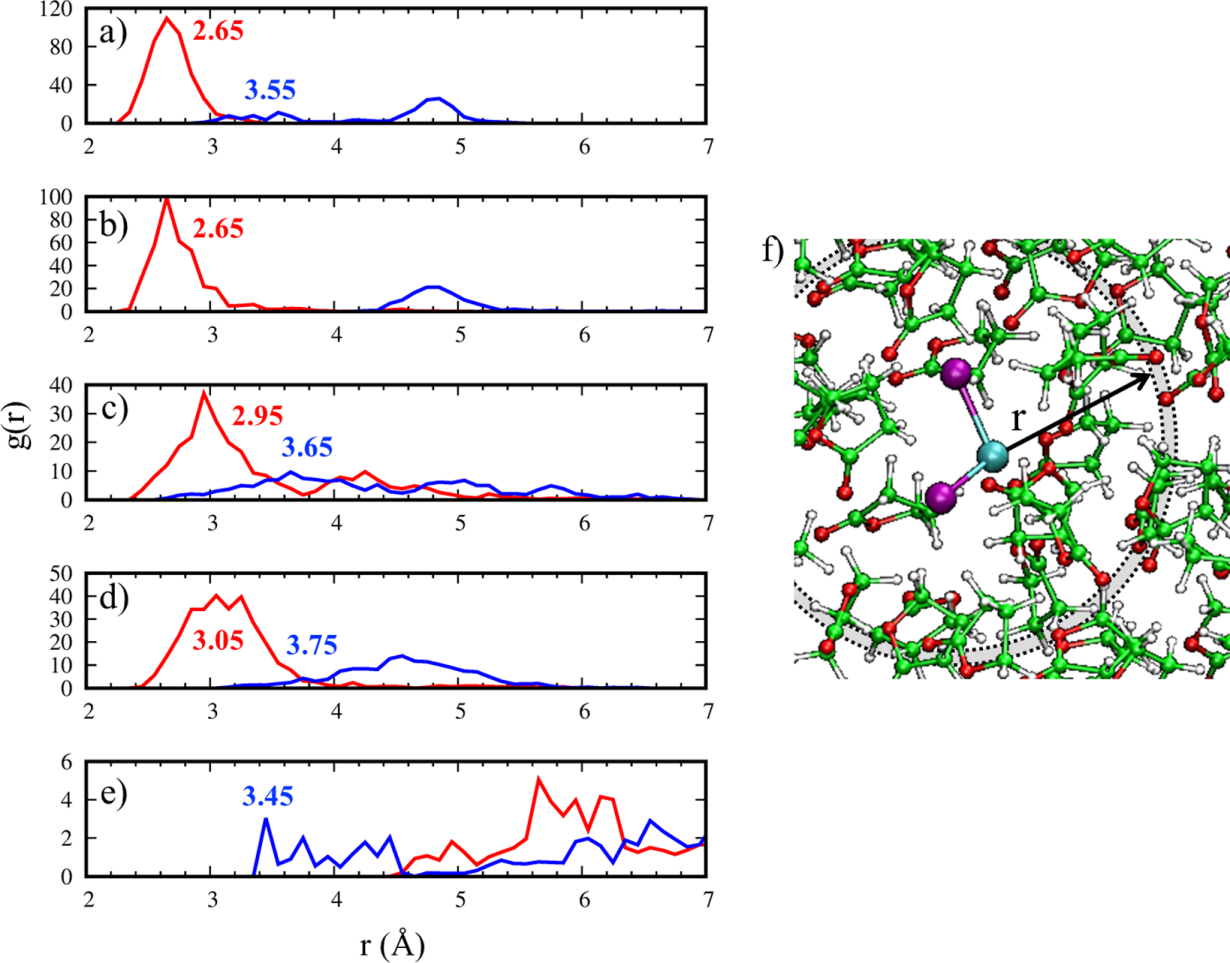
Panels a–e: *g*(*r*) of the Pb–O distance (*r*, Å) for the five most interacting GBL molecules considered in the molecular dynamics (red and blue lines correspond to carbonyl and alkoxy oxygens). Panel f: physical model employed to analyze the *g*(*r*) function.

On the basis of the information extracted from the AIMD simulation, we modeled PbI_2_ surrounded by a different number of coordinating GBL solvent molecules, thus considering the first coordination sphere, to evaluate the impact of the various coordination number and mode on the optical properties of solvated PbI_2_. In doing so, we considered both equatorial (eq) and axial (ax) PbI_2_ arrangements, adding an increasing number of GBL molecules, up to a maximum total coordination number of 7 (5 GBL and 2 I). The resulting nine optimized geometries are reported in Figure 3. In agreement with the outcome of AIMD simulation, we found that PbI_2_(GBL)_*n*_ complexes are stabilized by pincher-type interactions for *n* ≤ 4, while in the case of *n* = 5 the alkoxy oxygen is often too distant from the Pb center to interact, due to high steric hindrance. Again, in these complexes we can find average shorter carbonyl O–Pb distances with respect to the alkoxy O–Pb ones.

**Figure 3:**
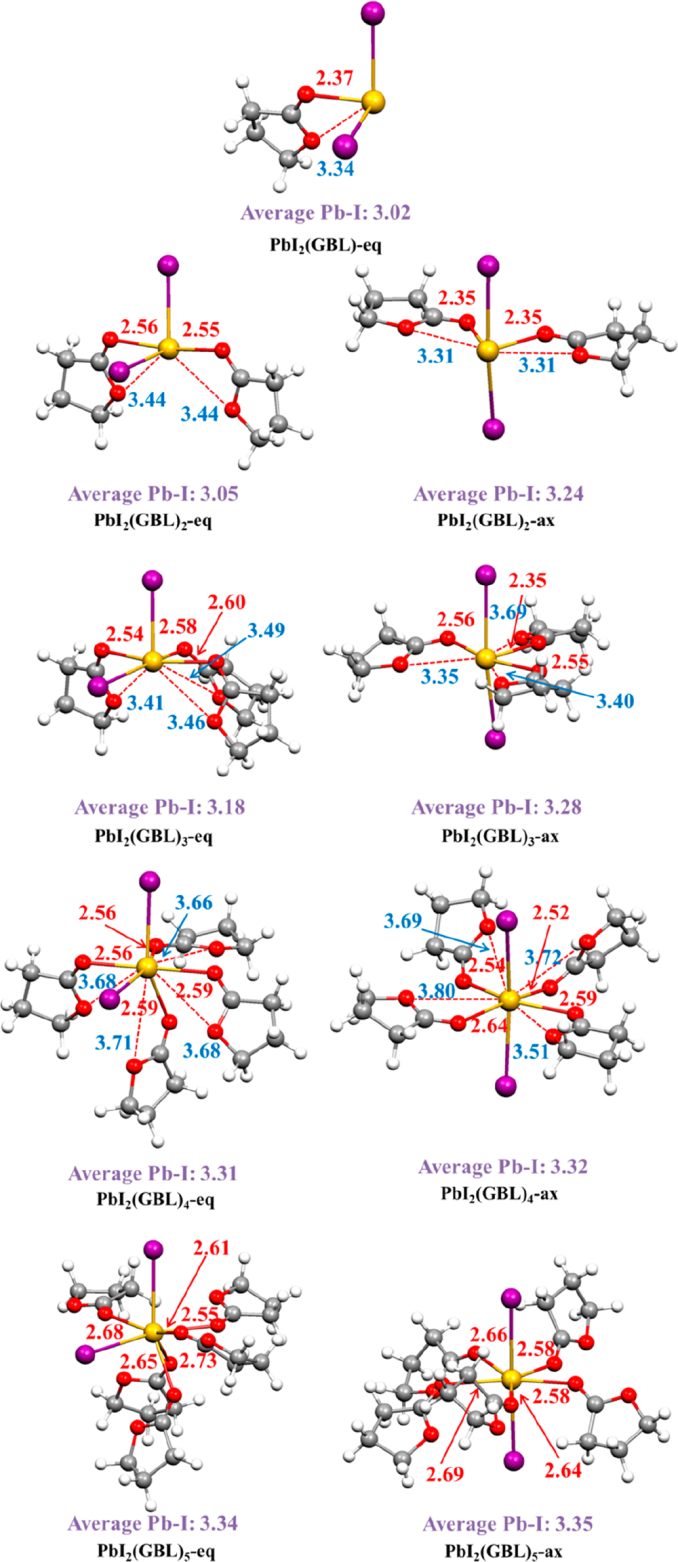
Optimized structure of PbI_2_(GBL)_*n*_ with *n* = 1–5 complexes. Main geometrical parameters are reported in Å (red and blue values correspond to carbonyl and alkoxy O–Pb bond lengths).

To clarify the effect of the solvent coordination on the optical properties of the PbI_2_(GBL)_*n*_ complexes (*n* = 1–5), we simulated the UV–vis absorption spectra with TD-DFT by employing relativistic spin–orbit coupling (SOC), by computing several excitation energies and oscillator strengths. The results are reported in Figure 4, together with the experimental absorption spectrum of PbI_2_ species in GBL solvent for a direct comparison. Due to the very low solubility of this salt, this spectrum was registered from the supernatant of a saturated solution of PbI_2_, taken after deposition of the undissolved PbI_2_ in order to avoid scattering signals. A summary of the first absorption maxima of each PbI_2_(GBL)_*n*_ complex, together with the relative energy of ax and eq configurations is reported in Table S1, Supporting Information.

**Figure 4:**
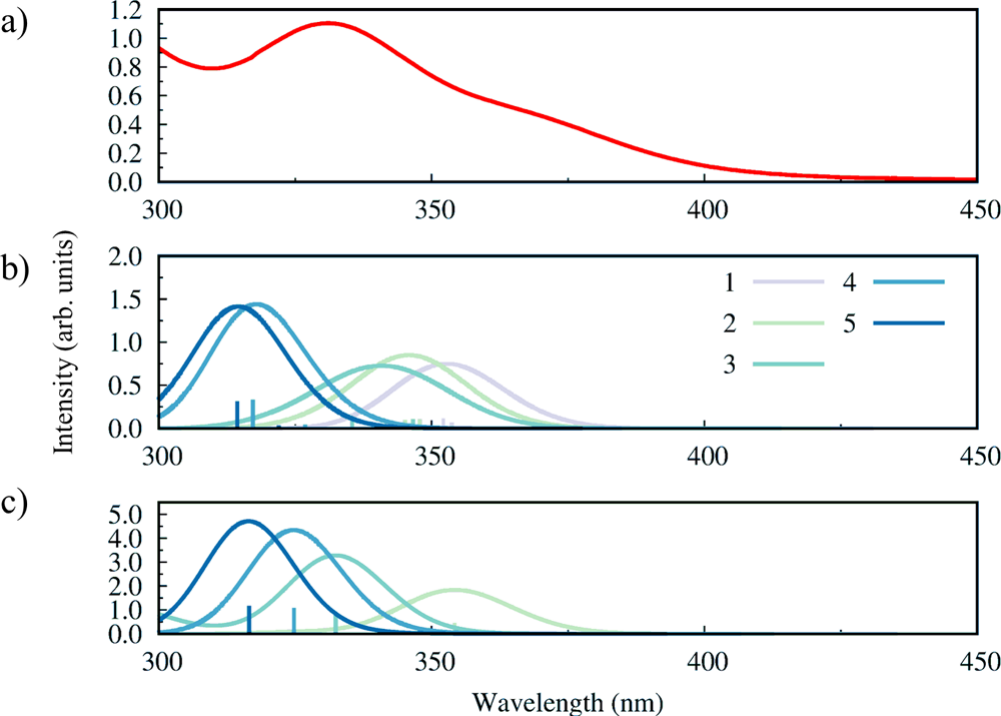
Experimental absorption spectrum of PbI_2_ in GBL (a) and theoretical absorption spectra of PbI_2_(GBL)_*n*_ complexes (eq and ax configurations in panels b and c, respectively), with the number of coordinating GBL molecules *n* reported in the legend.

At first glance, Figure 4 shows that the addition of GBL solvent molecules determines a monotonic blue shift of the first absorption peak, for both the ax and eq configurations, passing from 353 nm for the PbI_2_(GBL)-eq complex to 317/314 nm for the PbI_2_(GBL)_5_ complex in the ax/eq configuration. The solvated PbI_2_(GBL)_*n*_ complexes with *n* < 4 are found slightly energetically favored in the eq rather than in the ax configuration, consistent with the AIMD results. Interestingly, the first absorption maximum wavelength correlates quite well with the coordination number and with the average Pb–I and Pb–O bond length, as shown in Figure 5. In particular, we found that increasing the coordination, i.e., adding GBL molecules to the coordination sphere, is likely to blue-shift the absorption maximum. However, this occurs together with an increase in the average bond distances, both for Pb–I and Pb–O, which is expected when more coordinating molecules are added.

**Figure 5:**
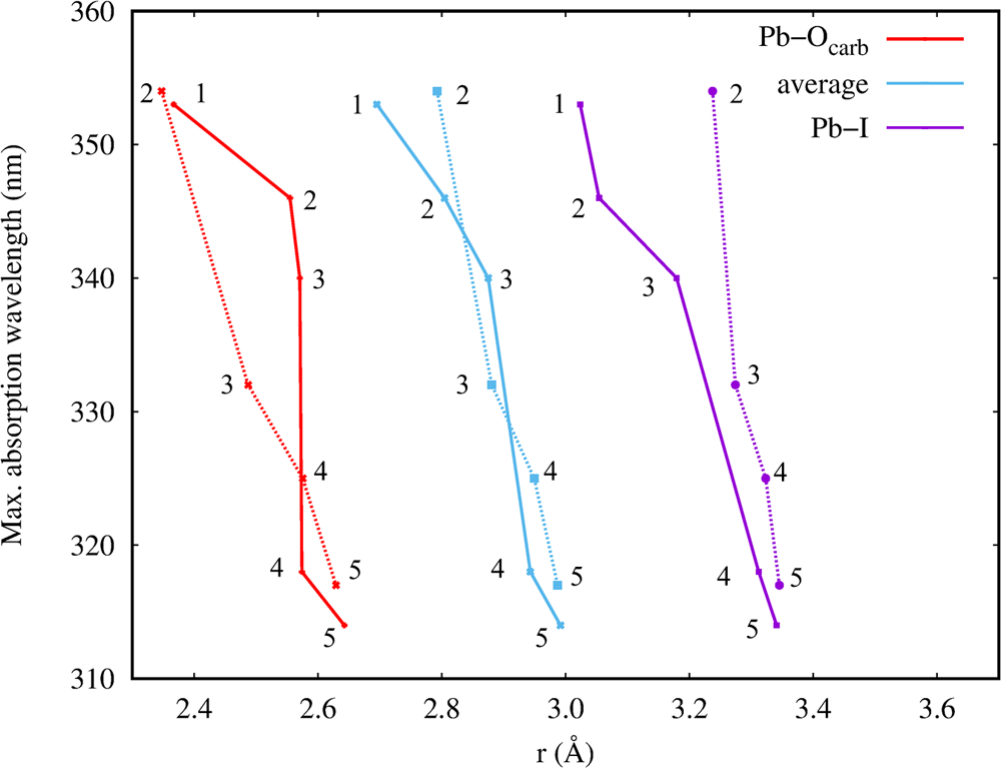
Average coordination/bond length–first absorption maximum correlation for the PbI_2_(GBL)_*n*_ complexes. Numbers in the picture point at the number of coordinating GBL molecules; solid and dashed lines refer to eq and ax configurations.

To further understand the relation between coordination and optical properties of PbI_2_ in GBL, we inspected how the absorption spectra change during the dynamics simulation. From the 15 ps of AIMD, we selected 20 snapshots (each 0.75 ps) and we carried out TD-DFT calculations with SOC effects included to simulate the absorption spectra, in the same way we did for the PbI_2_(GBL)_*n*_ complexes. For each snapshot we included only the GBL molecules highly interacting with the Pb ion, thus including only molecules for which the Pb–O solvation radius is lower than 4.0 Å and we considered only the first 12 excited states. These spectra were used to calculate a single time averaged spectrum (see Figure 6), which was then compared with the experimental UV–vis spectrum of PbI_2_ in GBL.

**Figure 6:**
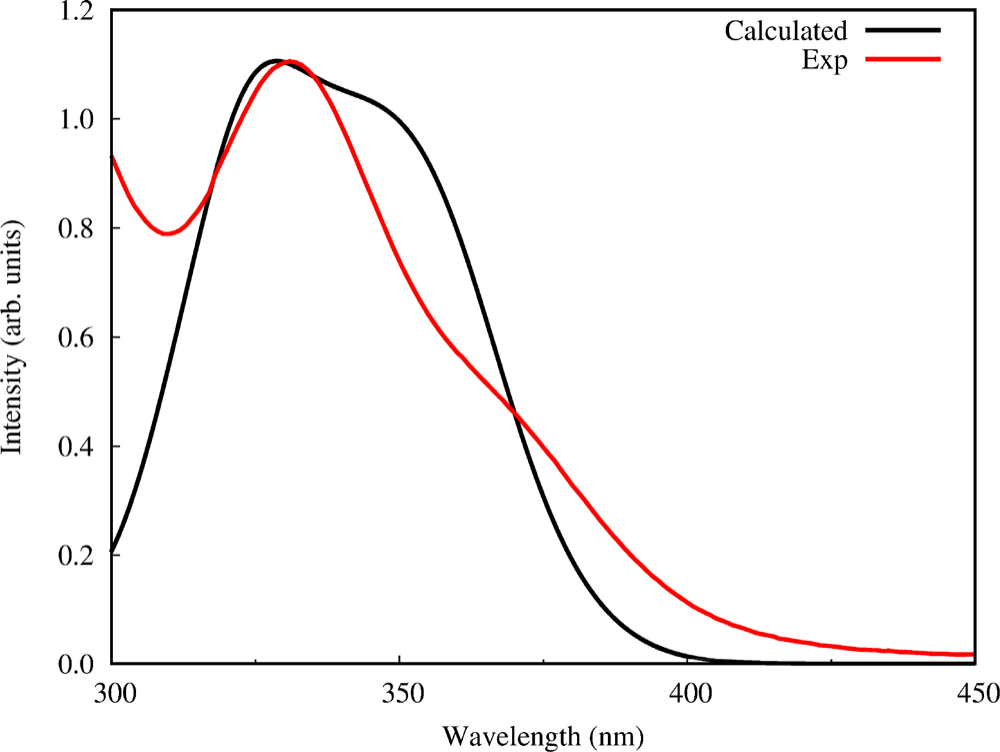
Comparison of the experimental (red) and time-averaged calculated (black) absorption spectrum of PbI_2_ in GBL. The intensity of the calculated spectrum was scaled to match that of the experimental one at the absorption maximum.

The agreement between the theoretical and experimental absorption spectra is eye-catching: the main experimental peak at 327 nm is adequately described by the time averaged spectrum, confirming the overall accuracy of the approach. Combining the information on the coordination obtained from AIMD and the evaluation of absorption spectra of PbI_2_(GBL)_*n*_ complexes, we can conclude that this absorption refers to the PbI_2_ species surrounded on average by three GBL molecules. The theoretical spectrum also features another band around 350 nm, which is mostly due to less intense coordination of PbI_2_ by GBL molecules, i.e., average longer Pb–O bond lengths. Interestingly, from the experimental spectrum we can notice a small absorption shoulder at 367 nm, that was assigned to the PbI_3_^–^ species both in GBL and in other solvents.^15,18,31−33^ This feature is not detected from our calculation, since at present we are not considering other iodoplumbates than PbI_2_.

It is finally interesting to make a comparison with the optical properties of the analogue PbI_2_(solv)_*n*_ complexes in DMSO and DMF (optimized structures reported in Figures S4 and S5, Supporting Information, respectively). In the case of the PbI_2_(DMSO)_5_ complex, we were not able to reach an energy minimum for the eq configuration, so that only the ax one is shown. As reported previously,^18^ the formation energies of iodoplumbates in GBL are always lower than those in DMF and DMSO, confirming the lower coordinative power of GBL that, as already said, reflects on the simulated spectra. In this paper, thanks to AIMD simulations, we found new stable structures for the PbI_2_(GBL)_*n*_ complexes compared to those evaluated in our previous paper,^18^ but this is not affecting the general coordination trend. In particular, taking into account common solvents employed in the perovskites synthesis, such as DMSO, DMF, GBL and ACN, we found that the coordination ability to PbI_2_ is in the order DMSO > DMF > GBL > ACN, in agreement also with experimental findings.^34,35^ The overall absorption spectra for the considered PbI_2_(solv)_*n*_ complexes in GBL, DMSO, and DMF solvents are reported in Figure S6, Supporting Information. In the DMF case, we can see the same trend encountered for the GBL spectra, moving from the less to the most coordinated complex, through both the ax and the eq series (see also Table S1, Supporting Information). However, as expected due to the higher coordinative power of DMF, the spectra are always blue-shifted compared to the case of GBL. In the case of DMSO, we find a similar situation to that of DMF. Interestingly, in this case there are some notable exceptions, represented by the spectrum of PbI_2_(DMSO)_4_-ax and PbI_2_ (DMSO)_2_-eq. This could be related to some particular interactions between the PbI_2_ and the DMSO molecules, which are not considered here.

First-principles simulations were carried out on a PbI_2_ model solvated in a common organic solvent used in the perovskite synthesis. Combining UV–vis spectroscopy and fine-tuned methodology developed in this work, we were able to characterize the local coordination of the PbI_2_ with GBL solvent. We found that PbI_2_ is coordinated on average by three GBL molecules, that are able to anchor through pincher-type interaction, i.e., involving both the carbonyl and the alkoxy oxygen atoms. However, these interactions are weaker than those established by higher coordinative solvents, such as DMSO or DMF, with direct consequences on many aspects, such as the solubility of precursors and solvent evaporation rate, that in turn influence the way perovskite grows, the morphology and the type of defects that will be found in the final material. The strength of the interactions plays a key role also on the description of the optical properties of these solutions. We found that simulated absorption spectra of PbI_2_(GBL)_*n*_, for a certain *n* number, are usually red-shifted with respect to their analogues in DMSO or DMF. Moreover, the wavelength of the first absorption maximum for each complex correlates quite well with bond lengths and coordination number. The excellent matching between the simulated absorption spectra and the experimental data confirms the reliability of our method for the study of diluted perovskite precursors solutions.

## Computational Details

AIMD simulations were carried out with the CP2K program,^36^ employing a DZVP basis sets, PBE functional,^37^ Goedecker–Teter–Hutter (GTH) pseudopotentials,^38,39^ Grimme van der Waals (vdW) correction (DFT-D3),^40^ and Cutoff = 550 Ry, Rel_Cutoff = 70 Ry. The time step of the integration of the dynamic equations was set to 0.5 fs. The VMD software^41^ was used to analyze changes in the solvent coordination sphere that occur during the dynamics. The molecular and electronic structure calculations on static complexes were performed with DFT using the Gaussian 09 software package,^42^ using the LANL2DZ basis sets for heavy Pb and also for I atoms together with the LANL2 pseudopotentials for the core electrons, while for light atoms (C, H, N, O, S) the 6-31G* basis sets were employed. The ADF 2014.04 program package^43−45^ has been used for the TD-DFT calculations, together with Slater type TZP basis sets for all the atoms (the cores 1s–2s, 1s–4p and 1s–4d were kept frozen, respectively, for S, I, and Pb) and a ZORA Hamiltonian to include SOC effects.^46,47^ In both cases we recurred to the B3LYP exchange–correlation functional.^48,49^ The absorption spectra have been simulated by interpolating the computed electronic transitions by Gaussian functions with a broadening σ = 0.1 eV. Solvents considered in this work were simulated both with implicit solvation models (polarizable continuum model, PCM in Gaussian09,^50^ and COSMO in ADF 2014.04^51^) and with explicit molecules, which are strictly necessary to investigate the specific chemical interactions, which are the main target of our study.

## Experimental Details

UV–vis absorption spectrum was recorded with a double beam spectrophotometer PerkinElmer Lambda 800, using quartz cuvettes with two optical walls and an optical path of 1 cm.

## Supporting Information Available

Figures of geometrical parameters and of theoretical spectra and table of first peak (highest wavelength) absorption maxima for the PbI_2_(solv)_*n*_ complexes (*n* = 1–5; solv = GBL, DMSO, DMF) (PDF)
