# Supplementary material for: Structural and Optical Properties of Solvated PbI2 in γ-Butyrolactone: Insight into the Solution Chemistry of Lead Halide Perovskite Precursors
Source: J Phys Chem Lett. 2020 Jul 9;11(15):6139–45. doi: 10.1021/acs.jpclett.0c01890 (PMC8009512; doi:10.1021/acs.jpclett.0c01890)
Supplement: Supplementary file 1 — jz0c01890_si_001.pdf [file jz0c01890_si_001.pdf]

Supporting Information

Structural and Optical Properties of Solvated PbI_2 in γ-Butyrolactone: Insight into the Solution Chemistry of Lead Halide Perovskite Precursors

Eros Radicchi,[1,2] Ali Kachmar,[3] Edoardo Mosconi,[2] Beatrice Bizzarri,[2] Francesca Nunzi,[1,2 *] and Filippo De Angelis[1,2,4 *]

[1]*Department of Chemistry, Biology and Biotechnology, University of Perugia, via Elce di Sotto 8, 06123 Perugia, Italy.*

[2]*Computational Laboratory for Hybrid/Organic Photovoltaics (CLHYO), Istituto CNR di Scienze e Tecnologie Chimiche "Giulio Natta" (CNR-SCITEC), via Elce di Sotto 8, 06123 Perugia, Italy.*

[3]*Qatar Environment and Energy Research Institute, Hamad Bin Khalifa University, Doha, Qatar.*

[4]*CompuNet, Istituto Italiano di Tecnologia, Via Morego 30, 16163 Genova, Italy.*

e-mail: francesca.nunzi@unipg.it, filippo@thch.unipg.it

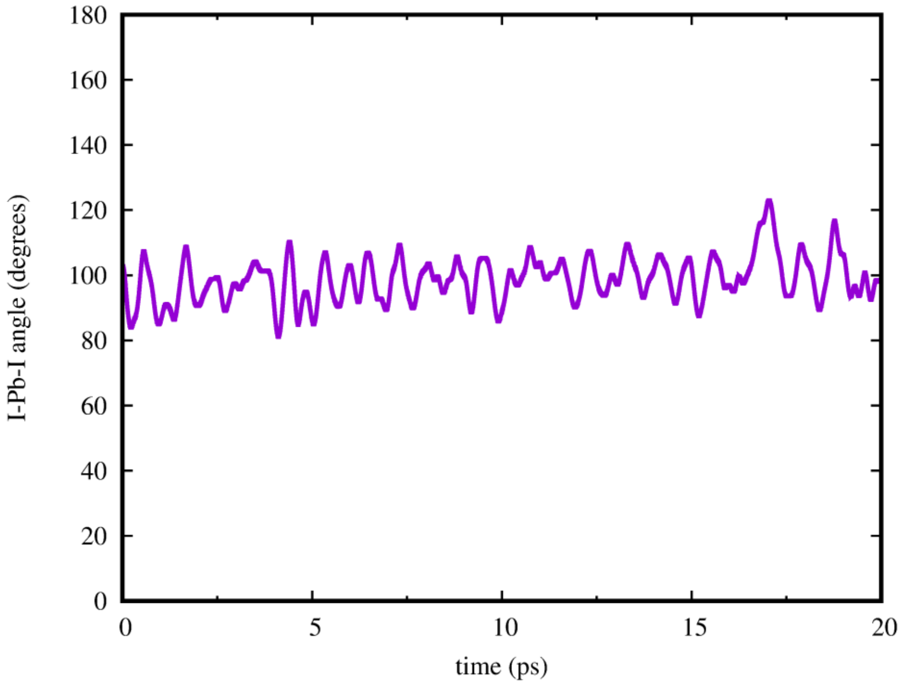

Figure S1. I-Pb-I angle analysis.

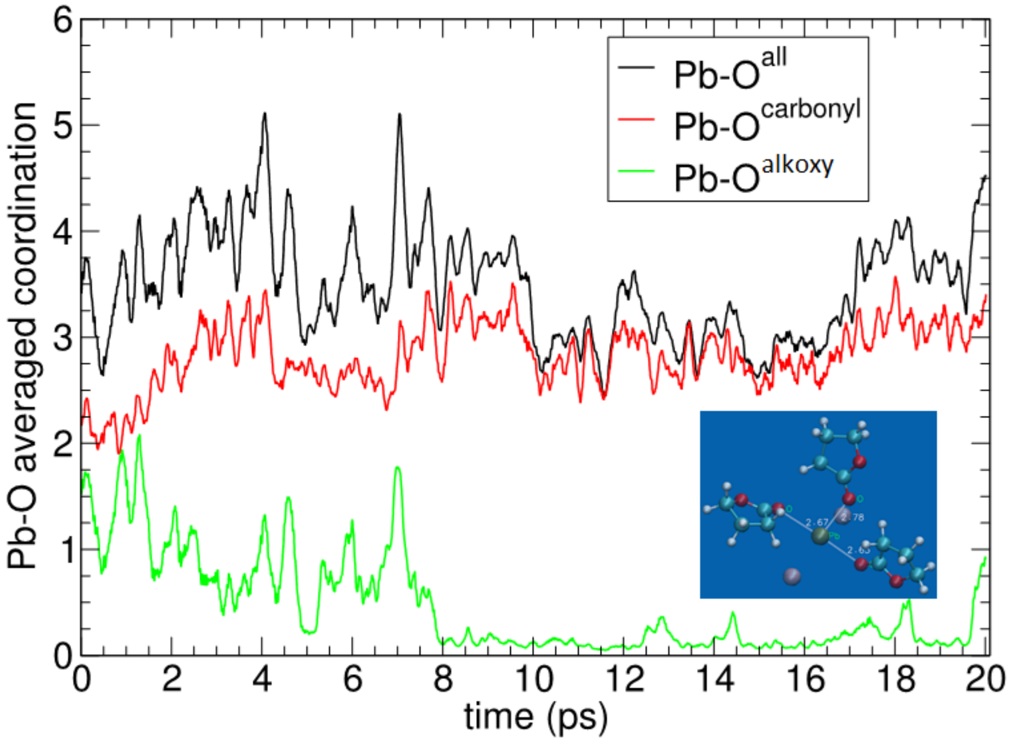

Figure S2. Average Pb-O coordination of the PbI_2 in GBL molecular dynamics simulation.

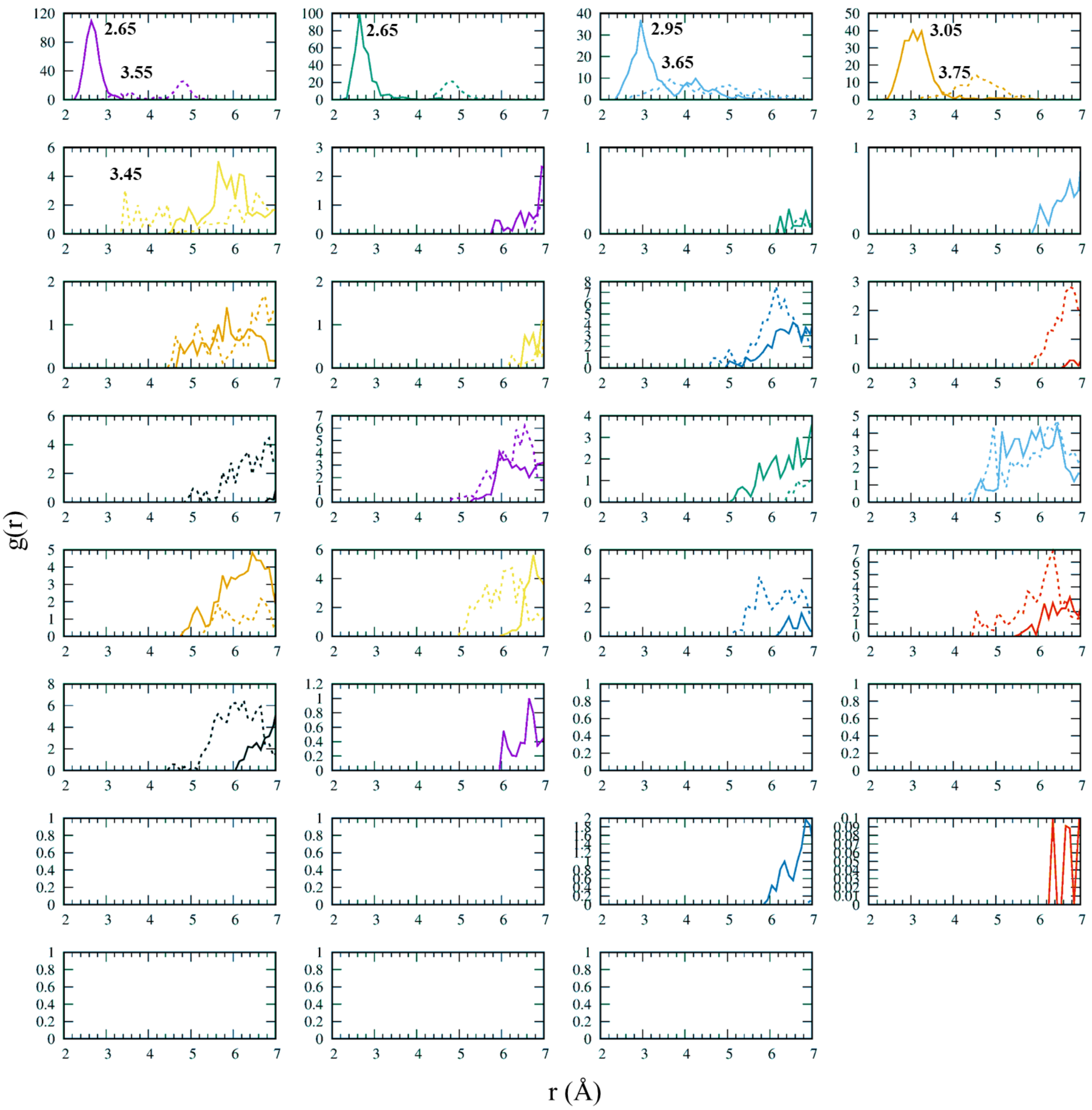

Figure S3. *g(r)* of the Pb – O distance (r, Å) for all the 31 GBL molecules considered in the molecular dynamics (continuous / dotted lines correspond to carbonyl / alkoxy oxygen).

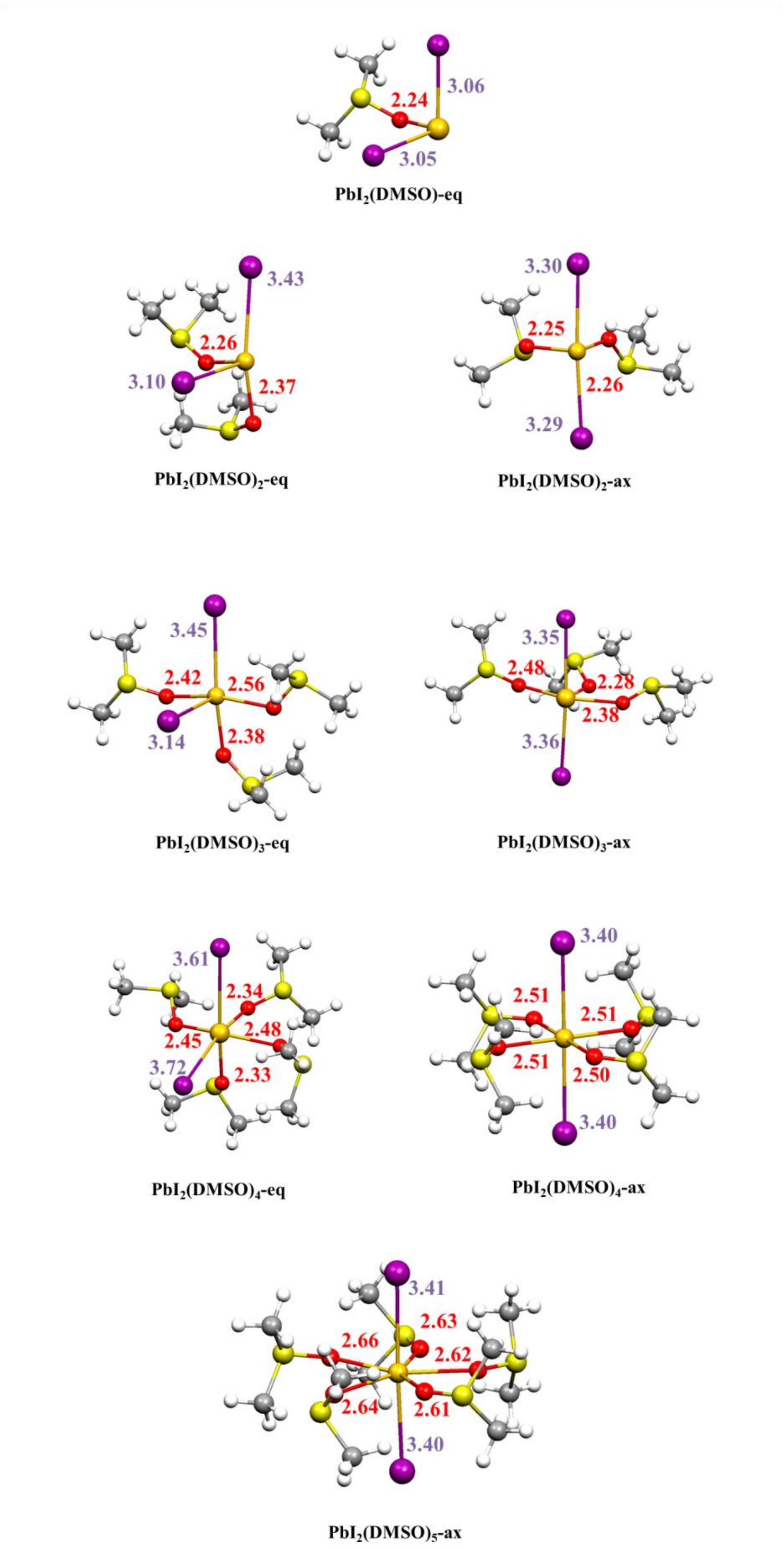

Figure S4. Optimized structure of $PbI_2(DMSO)_n$ with n = 1–5 complexes. Main geometrical parameters are reported in Å (red and purple values correspond to O−Pb and I−Pb bond lengths, respectively).

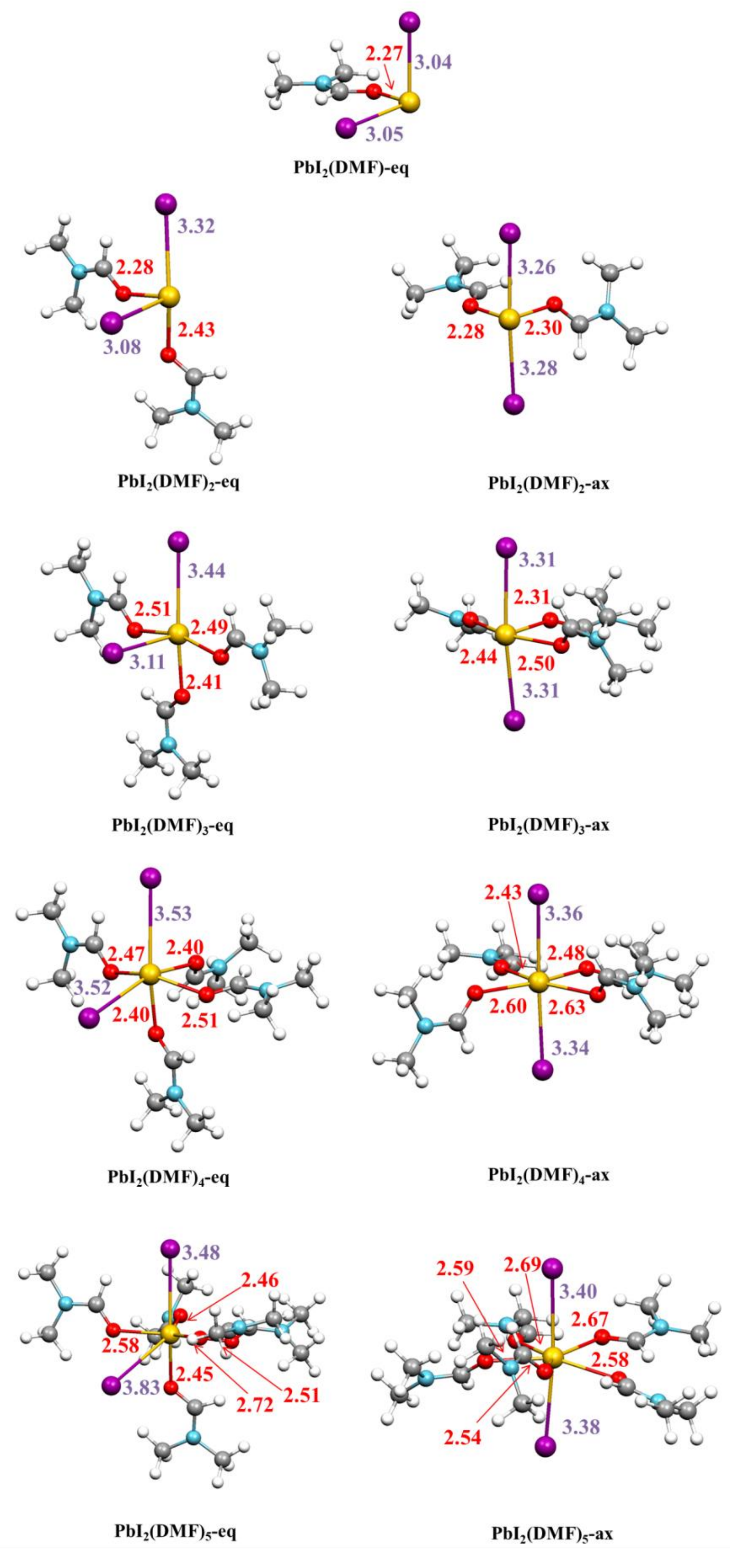

Figure S5. Optimized structure of $PbI_2(DMF)_n$ with n = 1–5 complexes. Main geometrical parameters are reported in Å (red and purple values correspond to O−Pb and I−Pb bond lengths, respectively).

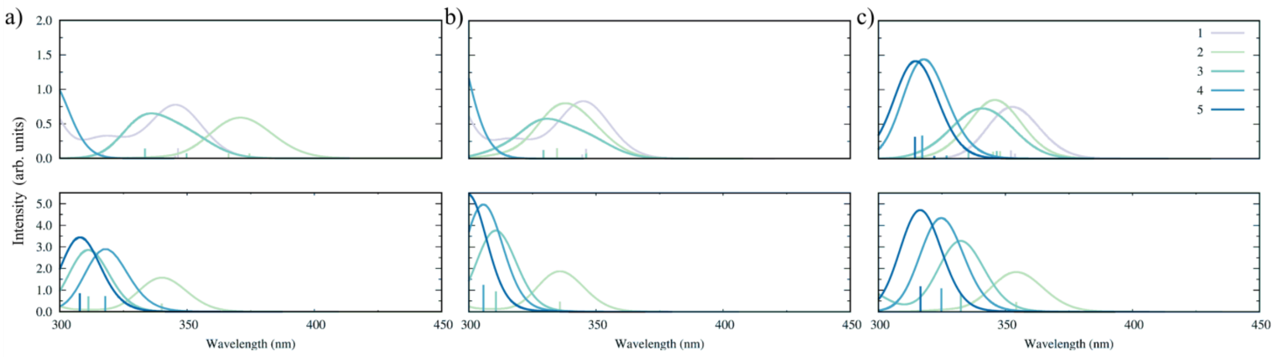

Figure S6. Theoretical $PbI_2(solv)_n$ spectra, for solv = a) DMSO, b) DMF and c) GBL, with values of n reported in the legend. Upper panel: eq configurations; lower panel: ax configurations.

n	DMSO			DMF			GBL		
	**λ, eq**	**λ, ax**	**ΔE**	**λ, eq**	**λ, ax**	**ΔE**	**λ, eq**	**λ, ax**	**ΔE**
1	345	/	/	345	/	/	353	/	/
2	370	340	-0.08	338	336	-0.07	346	354	0.07
3	336	311	-0.24	333	311	-0.16	340	332	0.00
4	295	318	0.12	292	306	0.10	318	325	-0.01
5	/	308	/		299	-0.06	314	317	-0.05

Table S1. First peak (highest wavelength) absorption maxima (nm units) of simulated absorption spectra and energy difference (eV units) between ax and eq configurations for $PbI_2(solv)_n$ (solv = DMSO, DMF, GBL and n = 1–5). The eq configuration is set as zero energy.
